# Measuring the cellular memory B cell response after vaccination in patients after allogeneic stem cell transplantation

**DOI:** 10.1007/s00277-020-04072-9

**Published:** 2020-06-09

**Authors:** Julia Winkler, Hannes Tittlbach, Andrea Schneider, Corinna Buchstaller, Andreas Mayr, Ingrid Vasova, Wolf Roesler, Michael Mach, Andreas Mackensen, Thomas H. Winkler

**Affiliations:** 1 Department of Internal Medicine 5, Hematology/Oncology, University Hospital Erlangen, Friedrich-Alexander-University Erlangen-Nuremberg, Glückstrasse 6, 91054 Erlangen, Germany; 2 Department of Biology, Division of Genetics, Nikolaus-Fiebiger-Center for Molecular Medicine, Friedrich-Alexander-University Erlangen-Nuremberg, Erlangen, Germany; 3 Department of Medical Informatics, Biometry, and Epidemiology, Friedrich-Alexander-University Erlangen-Nuremberg, Erlangen, Germany; 4 Department of Medical Biometry, Informatics and Epidemiology, University Hospital Bonn, Bonn, Germany; 5 Institute for Clinical and Molecular Virology, University Hospital Erlangen, Erlangen, Germany

**Keywords:** Memory B cells, Allogeneic stem cell transplantation, Vaccination after transplantation, Plasmablast

## Abstract

After allogeneic hematopoietic stem cell transplantation (HSCT), patients are repetitively vaccinated to reduce the risk of infection caused by the immune deficiency following allogeneic HSCT. By the vaccination of transplanted patients, the humoral memory function can be restored in the majority of cases. It is unknown, however, to what extent memory B cells derived from the donor contribute to the mobilization of antibody-secreting cells and long-term humoral memory in patients after allogeneic HSCT. We therefore analyzed patients after allogeneic HSCT for memory B cell responses 7 days after single vaccination against tetanus toxoid (TT), diphtheria toxoid (DT), pertussis toxoid (PT), *Haemophilus influenzae* type b (Hib), and poliovirus. Patients showed an insufficient mobilization of plasmablasts (PB) after vaccination, whereas healthy subjects (HD, *n* = 13) exhibited a significant increase of PB in the peripheral blood. Regarding vaccine-specific antibody-secreting PB, all HD responded against all vaccine antigens, as expected. However, only 65% of the patients responded with a measurable increase in IgG-secreting PB against TT, 65% against DT, 33% against PT, and 53% against poliovirus. Correspondingly, the antibody titers on day 7 after vaccination did not increase in patients. A significant increase of serum titers for the vaccine antigens was detectable in the majority of patients only after repetitive vaccinations. In contrast to the low mobilization of vaccine-specific PB after vaccination, a high number of PB before vaccination was detectable in patients following allogeneic HSCT. High frequencies of circulating PB correlated with the incidence of moderate/severe chronic GVHD. In summary, patients showed a weak mobilization of antigen-specific PB and an inadequate increase in antibody titers 7 days after the first vaccination. Patients with moderate or severe chronic GVHD in their history had a significantly higher percentage of IgG-secreting PB prior to vaccination. The antigen specificity of these IgG-secreting PB is currently unknown.

**Electronic supplementary material:**

The online version of this article (10.1007/s00277-020-04072-9) contains supplementary material, which is available to authorized users.

## Introduction

One of the major goals after allogeneic hematopoietic stem cell transplantation (HSCT) is to reconstitute the donor immune system in the patient. Immune reconstitution is defined as the restauration of the donor-derived pathogen-specific immunity. After HSCT, a long-lasting B cell deficiency is detectable, even when donor B cells are engrafted [1, 2]. The delayed B cell reconstitution leads to a persistent hypogammaglobulinemia and an increased rate of infections [3, 4]. This is mainly due to infections with viruses and encapsulated bacteria [5–8]. The rapid decline of antibody titers against vaccine-preventable diseases (e.g., tetanus, polio, measles, mumps, rubella) is a manifestation of this B cell deficiency following allogeneic HSCT when the recipient is not revaccinated [9–11].

It is known that reconstitution of B lymphocytes including memory B cells after allogeneic HSCT takes up to 2 years with transitional and naïve B cells dominating during the first year [12–15]. The cause for the long-lasting reduction of memory B cells, despite sufficient numbers of transitional and naïve B cells, is unknown and has been described as an IgM maturation block [16]. Eventually, the paucity of CD27^+^ memory B cells can lead to an inability to produce a proper B cell response to pathogens [17, 18]. The memory B cell response against vaccine antigens shows a very specific and fast mobilization of antigen-specific antibody-secreting cells (ASC) into the peripheral blood within 6 to 7 days [19]. ASCs are CD19^+^/CD27^high^/CD20^−^/CD38^high^-positive B cells corresponding to recently generated plasmablasts. These ASCs provide a short-lived peak antibody response and then either die or compete successfully for survival in bone marrow niches or in an inflamed tissue to provide long-lived humoral immunity [20].

As the B memory response to vaccine immunizations in patients after allogeneic HSCT is unknown, we intended to analyze the generation of antibody-secreting B cells and CD38^high^/CD27^high^ plasmablasts within 7 days after a single vaccination as an indicator of the status of the memory B cell compartment in patients after allogeneic HSCT.

## Methods

### Patients, healthy donors, and vaccination

Patient characteristics are summarized in Table 1. Between 2011 and 2016, 27 patients after d+180 of allogeneic HSCT were enrolled in the study approved by the institutional research ethics committee of the university Erlangen (Re. No. 147-12B). All patients provided informed consent.

**Table 1:** Patient characteristics and immunological parameters

Variables	No (%)
Number of patients	27
Age (years), median (range)	58 (18–74)
Gender	
Male/Female	20 (74)/7 (26)
Primary disease	
AML	18 (67)
MDS	6 (22)
Other (CML, MM, T-PLL)	3 (11)
Age of donors (years), median (range)	39 (19–60)
Donor type	
HLA-matched sibling	9 (33)
HLA-matched unrelated	18 (67)
Conditioning regimen	
FBM with CSA/MMF	20 (74)
Others with CSA/MTX (TBI/Cy, FLAMSA-RIC, Treo/Flu, Bu/Flu)	7 (26)
Rabbit ATG	
2.5 mg/kg b.w.	9 (33)
7.5 mg /kg b.w.	18 (67)
Day of vaccination after alloSCT, median (range)	226 (180–430)
Cellular and humoral parameters on the date of first vaccination	
CD3^+^ cells/μl, mean (range)	1103 (60–3006)
CD4^+^ cells/μl, mean (range)	241 (40–679)
CD19^+^ cells/μl, mean (range)	188 (16–758)
Plasmablasts/μl, mean (range)	8 (0–51)
Percentage of memory B cells of CD19+ cells/μl, mean (range)	11 (1–73)
Total IgG in g/l, mean (range)	7 (3–15)
Maximum grade of acute GVHD	
No/grade I	8 (31)
Grades II–IV	19 (69)
Maximum grade of chronic GVHD	
No/mild	17 (63)
Moderate/severe	10 (37)
Immunosuppressive therapy on the date of first vaccination	
Yes*	19 (69)
No	8 (31)

*Steroid therapy < 0.2 mg/kg and/or cyclosporine A (plasma level ≤ 50 ng/ml)

At the start of the vaccination, acute GVHD was resolved in all patients, and all patients with a chronic GVHD in their history had an inactive chronic GVHD and only a minimum dose of the immunosuppressive therapy (e.g., steroids, cyclosporine A). For treatment of chronic GVHD, exclusively steroids and cyclosporine A were given.

**Fig. 1:**
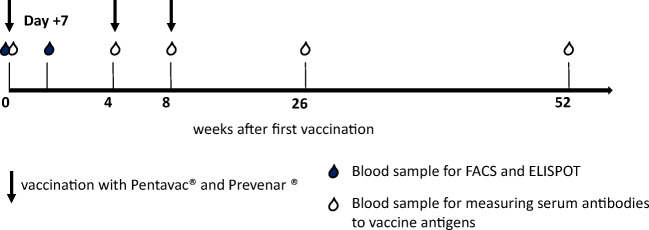
Schedule for vaccinations and blood sampling. Patients were vaccinated three times with Pentavac® and Prevenar 13® in an interval of at least 4 weeks. The blood samples for FACS analysis and for ELISPOT were done before and day + 7 and for detection of serum antibodies before and 4, 8, 26, and 52 weeks after the first vaccination

Exclusion criteria for vaccination were ongoing infections, disease relapse, immunosuppressive therapy with systemic steroid therapy > 0.2 mg/kg or with cyclosporine A > 50 ng/ml, administration of intravenous immunoglobulin in the 2 months prior to vaccination, and treatment with rituximab. Patients were vaccinated according to EBMT guidelines 3 times with an interval of at least 4 weeks (Fig. 1).

### Vaccines

The pentavalent combination vaccine PENTAVAC® (Sanofi Pasteur MSD GmbH) and the pneumococcal conjugate vaccine PREVENAR 13® (Wyeth Lederle Vaccines S.A.) were administered by intramuscular injection.

For comparison of the vaccine response, a group of volunteering healthy donors (*n* = 13, mean age 39 years, range 27–66) was vaccinated once with PENTAVAC®.

### Flow cytometry

Flow cytometry analysis was performed with a FACSCalibur instrument (Becton Dickinson, Heidelberg, Germany). All antibodies used are listed in the supplementary material (Table S1).

### Measurement of serum antibody titers by ELISA

IgG serum antibody titers were measured by using ELISA for tetanus toxoid (TT); diphtheria toxoid (DT); pertussis toxoid (PT); *Haemophilus influenzae* type b-polysaccharide (Hib); pneumococcal polysaccharide serotypes (pn) 1, 14, 23, and 26; and poliovirus serotypes 1, 2, and 3. For TT and DT (both obtained from Statens Serum Institut, Copenhagen, Denmark), and PT (Sigma) and Hib (HbO-HA, polysaccharide conjugated to human serum albumin, obtained from NIBSC, South Mimms, UK), ELISA 96-well plates (Greiner Bio-One GmbH) were coated with 5-μg/ml antigen. For antibodies against poliovirus, a commercial ELISA was used according to the instructions of the manufacturer (Demeditec Diagnostics GmbH, Kiel, Germany). The following WHO standards were used for calibration: TE-3 for TT, 10/262 for DT, 06/140 for pertussis, 09/222 for Hib, and 82/585 for poliovirus (NIBSC, South Mimms, UK). Protective antibody concentrations were defined as ≥ 0.1 IU/ml for TT and DT, ≥ 24 IU/ml for pertussis, ≥ 1 μg/ml for Hib, ≥ 10 U/ml for polio, and ≥ 0.35 μg/ml for pneumococcal polysaccharides. A positive response was defined as ≥ 4 times the minimum level of detection in the pre-vaccination sample (d+0) and ≥ 100% increase between the pre-vaccination sample (day 0) and the post-vaccination samples.

### Isolation of peripheral blood mononuclear cells and purification of B lymphocytes

Peripheral blood mononuclear cells (PBMCs) from patients and healthy donors were isolated from 80 ml of whole blood by Ficoll density gradient centrifugation (Lymphoflot®, Bio-Rad, Munich, Germany). After Ficoll separation, the PBMCs were washed, and untouched B cells were purified with a B Cell Isolation Kit II, human (Miltenyi Biotec, Bergisch Gladbach, Germany). The purity of the B cell preparations was determined by FACS analysis with CD19 antibodies for the calculation of input numbers in the enzyme-linked immuno spot (ELISPOT) assay.

### Quantification of antibody-secreting cells by enzyme-linked immuno spot assay

For the quantification of total and vaccine-specific IgG antibody-secreting cells, ELISPOT multiscreen plates (Millipore, Billerica, MA, USA) were directly coated with goat anti-human IgG, Fc specific (2.5 μg/ml, DIANOVA, Hamburg, Germany), TT (2.5 μg/ml), DT (2.5 μg/ml), pertussis (1:2.000, a kind gift from Sanofi Pasteur, Marcy l’Etoile, France), and Hib (1 μg/ml Hib oligosaccharide conjugated to human serum albumin, NIBSC, South Mimms, UK) in PBS overnight at 4 °C. Multiscreen plates were precoated with goat anti-poliovirus antibody followed by incubation of an inactivated polio vaccine preparation (types 1, 2, and 3), kindly provided by Sanofi Pasteur. After washing, plates were blocked with 200 μl RPMI/10% FCS at 37 °C. Purified B lymphocytes in different cell densities were incubated in 200 μl RPMI/10% FCS for 5 h at 37 °C. Plates were washed and incubated with HRP-goat antibody to human IgG (1:1.000, DIANOVA, Hamburg, Germany) overnight at 4 °C. ELISPOTs were detected by TMB substrate (KPL/Seracare, Milford, MA, USA) and analyzed using an ELISPOT reader and AID EliSpot v5.0 (AID Diagnostics, Strassberg, Germany).

### Statistical analysis

Comparison of means was performed using the Wilcoxon-Mann-Whitney test. For the analysis of the clinical predictors to the vaccination response, a multiple linear regression analysis was applied (likelihood ratio test). The threshold for the determination for a significant difference was set at *p* < 0.05.

## Results

### Decreased frequencies of memory CD27^+^ B cell subsets and increased frequencies of CD38^high^ CD27^high^ plasmablasts in transplanted patients before vaccination

**Fig. 2:**
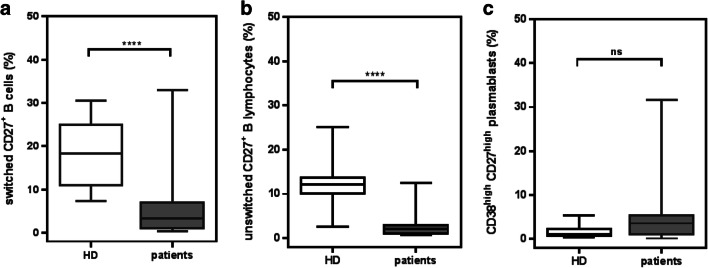
Frequencies of memory B cells and of plasmablasts in transplanted patients at the time point of the first vaccination. At the time point of the first vaccination (median 226 days after allogeneic HSCT), the frequencies of switched IgG^+^/CD27^+^ (**a**) and of unswitched IgD^+^/CD27^+^ (**b**) memory B cells are significantly reduced in patient at time of vaccination in comparison with healthy donors (HD). ****p < 0.0001 (Mann-Whitney test). The frequencies of CD27^high^/CD38^high^ plasmablasts (**c**) are increased at the same time point before vaccination. *p* = 0.089 (Mann-Whitney test)

Patients were vaccinated at a median of 226 days after allogeneic HSCT. The total numbers of circulating B lymphocytes were not significantly different in patients compared with healthy donors (HD, Supplementary Fig. 1A), reflecting an adequate reconstitution of B cells at this timepoint after HSCT. Patients revealed a significantly reduced frequency of CD27^+^/CD19^+^/CD38^low^ memory B cells compared with HD (Supplementary Fig. 1B). Both switched (IgD^−^; Fig. 2a) and non-switched (IgD^+^; Fig. 2b) memory B cell populations were significantly decreased in patients.

In contrast to the decreased memory B cell subsets, the frequency of CD38^high^/CD27^high^ plasmablasts was higher in the patient cohort in comparison with that in the HD, but this did not reach statistical significance for the overall patient cohort (Fig. 2c). In some patients, an extremely high percentage of plasmablasts up to 31.7% of all CD19^+^ cells was observed, which was not accompanied by an EBV or CMV reactivation.

### Insufficient mobilization of CD38^high^ CD27^high^ plasmablasts in HSCT patients after vaccination

**Fig. 3:**
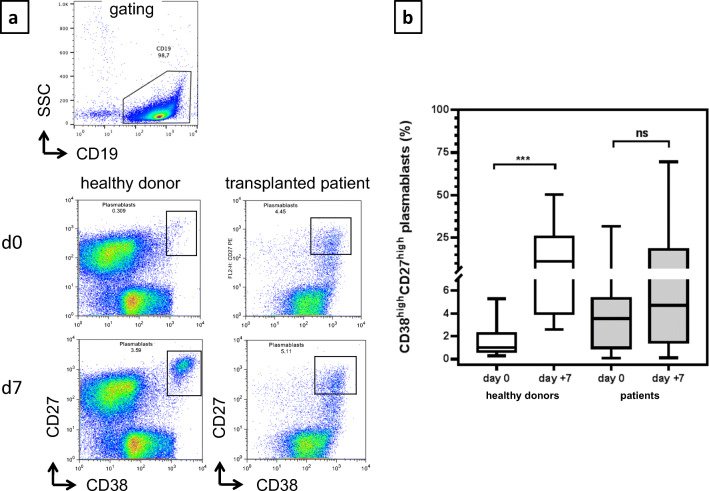
Mobilization of CD27^high^/CD38^high^ plasmablasts before and on d+7 after vaccination in patients and HD. **a** Mobilization with increase in frequencies of CD27^high^/CD38^high^ plasmablasts before vaccination and on day 7 after a single vaccination in an individual healthy donor and in an individual transplanted patient measuring by using flow cytometry. The gating strategy for the MACS-enriched B cells is shown. **b** Summarized data showing the frequency of CD27^high^/CD38^high^ plasmablasts in healthy donors (*n* = 10) and in patients (*n* = 27) before (d0) and 7 days (d+7) after vaccination. Patients (*n* = 27) showed an insufficient increase in CD27^high^/CD38^high^ plasmablasts (*p* = 0.15, Mann-Whitney test) in contrast to HD (***p = 0.0001, Mann-Whitney test)

It has been shown that CD38^high^ CD27^high^ plasmablast-secreting vaccine-specific IgG antibodies are mobilized in the peripheral blood 6–7 days after booster vaccination [19, 21]. These early appearing vaccine-specific plasmablasts are derived from memory B cells. As expected, on day + 7 after vaccination, we observed a significant increase of CD38^high^/CD27^high^ plasmablasts from median 1.0% of B cells to median 11.4% of B cells in HD (Fig. 3a, b). In contrast, we did not observe a significant increase of plasmablasts in patients on day + 7 (Fig. 3a, b). In contrast to the plasmablast response, the memory B cell subsets showed no increase both in HD and in patients on day + 7 after vaccination (data not shown).

**Fig. 4:**
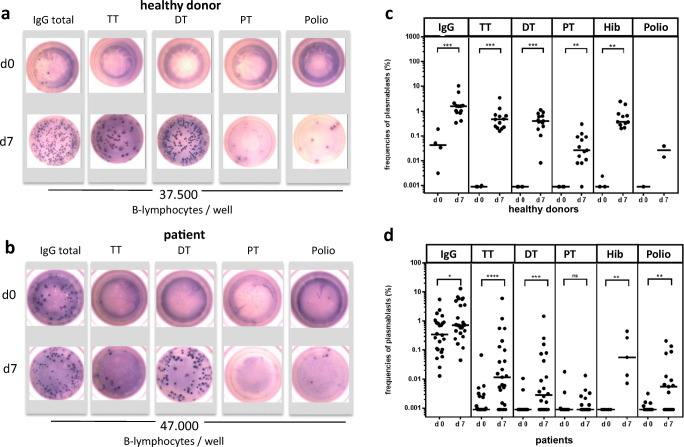
Frequencies of vaccine-specific plasmablasts enumerated than antibody-secreting cells (ASCs) by an ELISPOT before and on day + 7 after vaccination. **a** Example of the resulting spots of total IgG and of vaccine-specific ASCs (TT, DT, PT, Hib, poliovirus-specific ASCs before (d0) and 7 days (d7) after vaccination from an individual HD after seeding of 37,500 enriched B cells per well. **b** Example of the resulting spots of total IgG and of vaccine-specific ASCs d0 and d7 after vaccination from an individual patient after seeding 47,000 enriched B cells per well. **c** Frequencies of ASCs from HD d0 and d7 after vaccination. All HD responded with a significant increase of the frequency of vaccine-specific ASCs on d7. ***p = 0.0008 for total IgG ASCs, TT, and DT-specific ASCs, **p = 0.0038 for PT-specific ASCs, and **p = 0.0055 Hib-specific ASCs (Mann-Whitney test). Poliovirus-specific ASCs were tested only in two HD. **d** Patients’ frequencies of total IgG and vaccine-specific ASCs d0 and d7 after vaccination. Patients responded with a significant increase of the frequency for total IgG (*p = 0.034), TT (****p < 0.0001), DT (***p = 0.0002), Hib (**p = 0.0079), and poliovirus (**p = 0.0076) ASCs on d+7 (Mann-Whitney test). Not significant increase of frequency for PT-specific ASCs (*p* = 0.19) on d7 after vaccination in patients

To analyze the frequencies of vaccine-specific plasmablasts, we enumerated ASCs by using an ELISPOT technique. Examples of the resulting spots for a HD and a patient before and on day + 7 after vaccination are shown in Fig. 4a and b. A threshold of detection of 1/100,000 B cells seeded was set.

For TT, specific plasmablasts were undetectable (< 1/100,000) before vaccination, and a fulminant increase to a median frequency of 1/217 on day + 7 after vaccination was observed in all HD (Fig. 4c). In some patients, TT-specific ASCs were detectable before vaccination. However, a much more moderate increase to a median frequency of 1/8547 on day + 7 after vaccination was detected in the HSCT patients (Fig. 4d). Whereas all HD responded with an increase in frequency of TT ASCs on day + 7, only 65.2% (15/23) patients responded with an increase of TT ASCs.

For DT, the frequencies of DT-specific ASCs were undetectable before vaccination and revealed a significant increase to a median frequency of 1/253 on day + 7 after vaccination in HD (Fig. 4c). In allogeneic HSCT patients, the frequencies of DT-specific ASCs showed a lower increase from ≤ 1/100,000 before to 1/35714 on day + 7 after vaccination. Whereas all HD responded with a significant increase in frequency of DT ASCs, only 65% (13/20) responded in the patient cohort (Fig. 4d).

For pertussis, ASCs were undetectable in HD before vaccination and a rise in frequency to a median of 1/3745 on day + 7 after vaccination was observed. In the patient cohort, no significant increase in the median frequency was detectable on day + 7 (Fig. 4d). Whereas all HD except one individual responded against PT, only 33.3% (5/15) of the patients showed an increase of pertussis ASCs.

For Hib, specific ASCs increased to a median frequency of 1/272 on day + 7 in HD. In patients, the frequency of Hib-specific ASCs increased to a median of 1/1779 on day + 7 after vaccination. The percentage of responders was 75% (3/4) in HD and 100% (5/5) in patients.

For poliovirus, only two HDs could be analyzed. Poliovirus-specific ASCs increased to 1/3759 on day + 7 after vaccination. In patients, a median frequency of 1/17857 on day + 7 after vaccination was measured. A total of 8/15 (53.3%) patients showed significant responses.

In addition, we found a significantly higher frequency of IgG-secreting ASCs in patients in comparison with HD before vaccination (median 1/287 in patients vs. median 1/2347 in HD, *p* = 0.01). Whereas in HD, IgG ASCs increased approximately 40 fold to a median frequency of 1/64 on day + 7 after vaccination (Fig. 4c), patients showed only a twofold increase to a median frequency of 1/137 (Fig. 4d).

In summary, we observed robust plasmablast responses in almost all HDs for all vaccine antigens, resulting in high frequencies of ASCs on day + 7 after booster vaccination. In allogeneic HSCT patients, the magnitude of the response was considerably lower, and a large fraction of patients did not show any detectable ASC response.

### Absence of a measurable serum response in allogeneic HSCT patients on day + 7 after vaccination

**Fig. 5:**
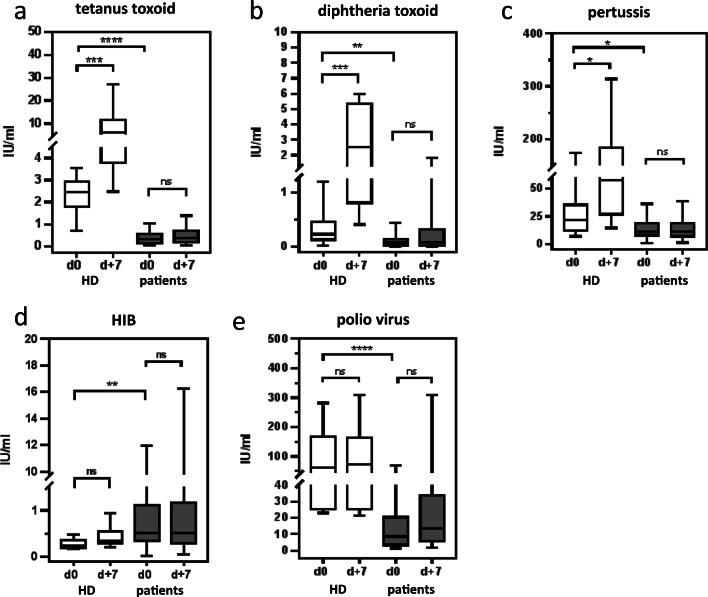
Serum titer against vaccine-specific antigens before and 7 days after single vaccination in HD and in patients. **a**–**e** The serum titers before vaccination (d0) and on day +7 after single vaccination from HD (white) and patients (black) by testing with the Mann-Whitney test. In HD, a significant increase of serum titer on d+7 after vaccination against TT (**a** ***p = 0.0003), DT (**b** ***p = 0.0002), and PT (**c** *p = 0.036) and a not significant increase of serum titer against Hib (**d***p* = 0.13) and poliovirus (**e***p* = 0.93) was detectable. In patients, a not significant increase of serum titer was shown on d+7 after vaccination against all vaccine antigens (**a** TT, *p* = 0.41; **b** DT, *p* = 0.46; **c** PT, *p* = 0.86; **d** Hib, *p* = 0.89; **e** poliovirus *p* = 0.19). Before vaccination, the serum titer was significantly reduced against all vaccine antigens in patients compared with HD. **a** TT serum titer (****p = < 0.0001). **b** DT serum titer (**p = 0.0036). **c** PT serum titer (*p = 0.028). **d** Hib serum titer (**p = 0.007). **e** poliovirus titer (****p < 0.0001)

Measuring the vaccine titer, we found considerably lower titers for TT, PT, DT, and poliovirus in HSCT patients prior to vaccination compared with HD (Fig. 5a–c, e). Interestingly, the Hib antibody titer was significantly higher in patients (*p* < 0.05). Only a fraction of patients had low protective titers against the vaccine antigens (88% for TT, 42% for DT, 8% for PT, 33% for Hib, 46% for poliovirus, 67% for pn1, 83% for pn14, 54% for pn23, 46% for pn26) (Table 2). HD showed a significant increase of serum titers for most antigens (*p* < 0.05) on day + 7 after booster vaccination, except for poliovirus, for which already high serum titers were measured before vaccination (Fig. 5e). HSCT patients, however, did not show a significant increase of antibody titer for any of the vaccine antigens on day + 7 after the first vaccination in serum (Fig. 5a–e), supporting the data for the low increase in vaccine-specific plasmablasts in the peripheral blood.

**Table 2:** Number of vaccine responders and number of patients with protective titer

Time (weeks)	0	4	8	26	52
Number of patients tested (*n*)	24	18	18	23	19
TTd No (%)					
Responder	-	13 (72)	16 (89)	22 (96)	19 (100)
Protective titer	21 (88)	18 (100)	18 (100)	23 (100)	19 (100)
DTd No (%)					
Responder	-	7 (39)	12 (67)	20 (87)	18 (95)
Protective titer	10 (42)	13 (72)	17 (94)	22 (96)	18 (95)
PTd No (%)					
Responder	-	10 (55)	14 (78)	19 (83)	18 (95)
Protective titer	2 (8)	8 (44)	15 (83)	21 (91)	18 (95)
HIB No (%)					
Responder	-	10 (56)	12 (67)	19 (83)	17 (90)
Protective titer	8 (33)	12 (67)	16 (89)	19 (83)	17 (90)
Poliovirus No (%)					
Responder	-	2 (11)	3 (17)	6 (26)	11 (58)
Protective titer	11 (46)	10 (56)	8 (44)	12 (52)	14 (74)
Pneumococcus serotype 1 No (%)					
Responder	-	10 (55)	8 (44)	17 (74)	13 (68)
Protective titer	16 (67)	13 (72)	13 (72)	22 (96)	15 (79)
Pneumococcus serotype 14 No (%)					
Responder	-	10 (55)	11 (61)	15 (65)	12 (63)
Protective titer	20 (83)	18 (100)	18 (100)	23 (100)	19 (100)
Pneumococcus serotype 23 No (%)					
Responder	-	12 (67)	14 (78)	21 (91)	12 (63)
Protective titer	13 (54)	13 (72)	18 (100)	23 (100)	19 (100)

### Serological response in allogeneic HSCT patients after repetitive vaccination

**Fig. 6:**
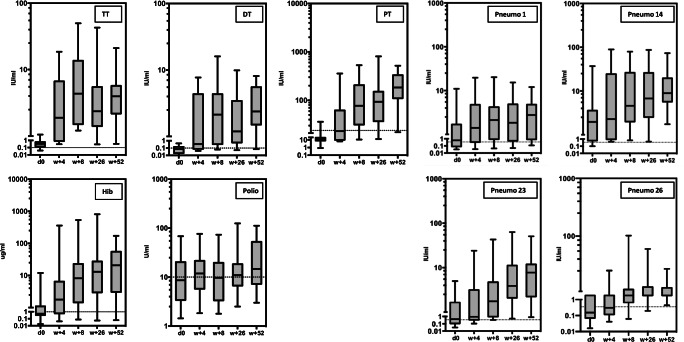
Serum response after three repetitive vaccinations in patients. The serum titer against the individual vaccine antigens after three vaccinations before (d0) and 4, 8, 26, and 52 weeks after vaccinations in patients. The dotted line indicated the protective titer of the individual vaccine antigen. For the week after vaccination, a protective serum titer was achieved in 100% of pts. for TT, in 72% of pts. for DT, in 44% of pts. for PT, in 67% of pts. for Hib, in 56% of pts. for poliovirus, in 72% of pts. for pneumococcus serotype 1, in 100% of pts. for pneumococcus serotype 14, in 72% of pts. for pneumococcus serotype 23, and in 44% of pts. for pneumococcus serotype 26

The schedule for repetitive vaccinations and serum samples is shown in Fig. 1. The serological responses are summarized in Fig. 6 and Table 2. For all antigens except polio, the antibody titers before vaccination were below or only marginally above protective titers. Variable vaccine responses were achieved 4 weeks after the first vaccination. The median antibody titers, the percentage of responders, and the percentage of patients having achieved a protective antibody titer increased over time and recurrent vaccinations for all antigens, with the interesting exception of poliovirus. Protective antibody titers were achieved for almost all patients 1 year after the start of the vaccination.

### Association of baseline immunological data and clinical parameters to vaccine plasmablast responses

We intended to find possible predictors for the highly variable plasmablast responses in vaccinated patients. As shown in Table 3, the seronegative donor and recipient CMV status (R0D0), the donor age, the number of CD3-positive and CD4-positive T cells, and the number of CD27^+^ memory B cells had a significant predictive value for the response in the ELISPOT against TT.

**Table 3:** Predictors for tetanus toxoid-IgG-secreting cells

Variable	*B*	*p* value	Lower 95% CL	Upper 95% CL
Dose level of ATG 2.5 vs. 7.5 mg/kg b.w.	0.6348	0.192	− 0.3440	1.6135
CMV serostatus R1D1, R1D0, R0D1 vs. R0D0	− 1.1521*	<0.01	− 1.9779	− 0.3263
Grade of acute GVHD	0.5205	0.287	− 0.4720	1.5130
Grade of chronic GVHD	0.8637	0.054	− 0.0147	1.7421
Recipient age	0.0001	0.995	− 0.0310	0.0312
Donor age	0.0469	< 0.05	0.0126	0.0812
Day of start vaccination	0.0064	0.074	− 0.0007	0.0136
Number of CD3^+^ T cells	0.0007	< 0.01	0.0002	0.0012
Number of CD4^+^ T cells	0.0041	< 0.01	0.0018	0.0064
Number of CD19^+^ B cells	− 0.0010	0.458	− 0.0038	0.0018
Percentage of CD27^+^ memory B cells	0.0860	< 0.001	0.0425	0.1295
Serum IgG concentration	0.0859	0.339	− 0.0975	0.2692

Multiple regression analysis for the estimation of relationship between of the frequencies of tetanus toxoid-secreting B cells d+7 after a single vaccination and transplant and immunological parameters before vaccination

*Recipients with CMV serostatus R0D0 had the lower frequency of anti-TT-IgG-producing B lymphocytes in comparison with CMV status R1D1, R1D0, R0D1

*B*, regression coefficient; *CL*, confidence limit

Interestingly, the ATG dose, the age of recipient, and the history of acute or chronic GVHD were no predictors for higher frequencies of TT-producing B cells in the ELISPOT assay.

### High percentages of IgG-secreting plasmablasts in patients with chronic GVHD before vaccination

**Fig. 7:**
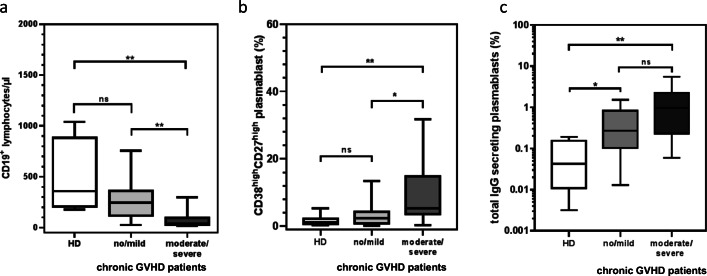
Comparison of frequency of B lymphocytes, CD38^high^/CD27^high^ plasmablasts, and spontaneously IgG-secreting B cells in patient with GVHD compared with HD. **a** The number of B cells was lowest in patients with moderate/severe GVHD. **p = 0.0012 in pts. with moderate/severe GVHD compared with pts. with no/mild GVHD, **p = 0.007 in pts. with moderate/severe GVHD compared with HD. **b** The frequencies of CD38^high^/CD27^high^ plasmablasts were the highest in patients with moderate/severe GVHD. *p = 0.027 in pts. with moderate/severe GVHD compared with pts. with no/mild GVHD, **p = 0.0076 in pts. with moderate/severe GVHD compared with HD. **c** The frequencies of IgG-secreting B cells were the highest in patients with moderate/severe GVHD compared with HD. **p = 0.0.0081. No/mild chronic GVHD patients had a significantly higher frequency of IgG-secreting B cells compared with HD. *p = 0.048. All statistical test were performed with the Mann-Whitney test

Patients with a moderate or severe chronic GVHD revealed the lowest number of B lymphocytes compared with patients with no or mild chronic GVHD (Fig. 7a). Interestingly, patients with moderate/severe chronic GVHD in their history had a significantly elevated frequency of plasmablasts (Fig. 7b). The frequency of IgG-secreting B cells was significantly increased in patients without or with only mild forms of chronic GVHD as well as patients with moderate/severe chronic GVHD as compared with HDs (Fig. 7c).

## Discussion

In this study, we investigated for the first time the generation of antibody-secreting plasmablasts in patients after allogeneic SCT in response to a single vaccination specifically to study the contribution of memory B cells derived from the donor. It is a specific feature of memory B cells that they can form Ag-specific plasmablasts after booster vaccination appearing in a wave in the peripheral blood already 6 to 7 days after vaccination [19]. Opposite to the robust mobilization of plasmablasts and vaccine-specific IgG, ASCs in HD patients after allogeneic HSCT exhibited a much weaker increase of the frequency of plasmablasts on day + 7 after vaccination, despite a comparable age of donors and volunteers from the control group. In addition, the increase in plasmablast frequency on day + 7 after vaccination varied substantially in individual patients and ranged from undetectable plasmablast mobilization to strong plasmablast mobilization in a few individuals. Correspondingly, the increase in frequency of vaccine-specific ASCs on day + 7 after vaccination was generally low, particularly against PT. Interestingly, the ASC response against Hib was comparable to HD, suggesting immunization by bacterial infections following allogeneic HSCT.

Altogether, these findings indicated that the reactivation of vaccine-specific memory B cells in patients after allogeneic HSCT is diminished with a high degree of variability among patients. Low frequencies of memory B cells are one explanation for the attenuated plasmablast response, and we confirmed that low frequencies are also found in previous publications (Fig. 2) [12, 22, 23]. This is supported by a correlation of the frequency of CD27^+^ memory cells with the d+7 plasmablast response against TT in our patients. However, other factors influence the early plasmablast response. First, the frequency of CD4^+^ T cells correlated with the plasmablast response against TT, indicating a T helper cell-dependent memory response against TT. A positive correlation with the age of the donor furthermore suggests a better vaccination status among older donors. Importantly, the dose of ATG and the severity of previous acute and chronic GVHD had no influence on the plasmablast response. Our finding that the CMV status of the donor or recipient is associated with significantly higher plasmablast responses is interesting in the light of recent findings of Furman et al., showing that CMV-seropositive young adults exhibited enhanced antibody responses to influenza vaccination [24].

The origin of memory B cells in patients after allogenic HSCT is therefore an important question for the understanding of memory B cell biology in these patients. Unselected peripheral stem cell preparations contain high numbers of memory B cells that are transferred to the recipient [2]. These memory cells from the vaccinated donor most likely give rise to the antigen-specific plasmablast response on day 7. It remains to be analyzed in the future what parameters allow survival and/or reactivation of theses donor-derived memory B cells only in some patients. Treatment with ATG might be one factor influencing the number of memory B cells surviving in the recipient. It has been shown that the presence of B cell reactive antibodies in the ATG preparations can deplete B cells as well as plasma cells [25]. The recovery of CD19^+^ B cells was significantly delayed in patients with allografts from unrelated donors receiving ATG as compared with patients with allografts from a matched family donor which had no ATG [26]. Importantly, however, adoptive transfer of additional memory B cells from the donor after transplantation might be a promising approach to lower the risk of post-transplant infections.

We performed three repetitive vaccinations in the patient cohort as suggested by the EBMT guidelines [27]. After these consecutive vaccinations, most patients exhibited protective antibody titers. Our detailed quantitative analysis of antibody titers extends previous findings [27–29] and confirms the efficacy of the vaccination regimen in patients after allogeneic HSCT.

In contrast to the low numbers of CD27^+^ memory B cells and to low mobilization of plasmablast after vaccination, we found a high frequency of plasmablasts in patients before vaccination. Elevations of spontaneous Ig-secreting plasmablasts have been described for patients with active chronic GVHD [30, 31] and also for patients with active systemic lupus erythematodes (SLE) [32]. The elevated plasmablast frequency is a sign of a general dysregulation of the B cell compartment that is associated with chronic GVHD as reviewed recently [33]. The specificity of the antibodies secreted by these plasmablasts remains elusive, however.

In summary, the weak mobilization of plasmablasts and the lack of serum response on day 7 after antigen contact by a booster vaccination illustrated the immunodeficiency produced by allogeneic HSCT. Instead, a dysregulation of the functional B cell response with high frequencies of plasmablasts can be observed in patients after allogeneic HSCT. The specificity of the antibodies produced by the plasmablasts remains to be investigated and could contribute to the understanding of the pathogenesis of chronic GVHD.

## Electronic supplementary material

ESM 1 (PDF 90 kb).

ESM 2 (DOCX 62 kb).

## References

[1] Petersen SL, Ryder LP, Björk P, Madsen HO, Heilmann C, Jacobsen N, Sengeløv H, Vindeløv LL (2003). A comparison of T-, B- and NK-cell reconstitution following conventional or nonmyeloablative conditioning and transplantation with bone marrow or peripheral blood stem cells from human leucocyte antigen identical sibling donors. Bone Marrow Transplant.

[2] Storek J, Dawson MA, Storer B, Stevens-Ayers T, Maloney DG, Marr KA, Witherspoon RP, Bensinger W, Flowers ME, Martin P, Storb R, Appelbaum FR, Boeckh M (2001). Immune reconstitution after allogeneic marrow transplantation compared with blood stem cell transplantation. Blood.

[3] Podgorny PJ, Pratt LM, Liu Y, Dharmani-Khan P, Luider J, Auer-Grzesiak I, Mansoor A, Williamson TS, Ugarte-Torres A, Hoegh-Petersen M, Khan FM, Larratt L, Jimenez-Zepeda VH, Stewart DA, Russell JA, Daly A, Storek J (2016). Low counts of B cells, natural killer cells, monocytes, dendritic cells, basophils, and eosinophils are associated with postengraftment infections after allogeneic hematopoietic cell transplantation. Biol Blood Marrow Transplant.

[4] Storek J, Espino G, Dawson MA, Storer B, Flowers ME, Maloney DG (2000). Low B-cell and monocyte counts on day 80 are associated with high infection rates between days 100 and 365 after allogeneic marrow transplantation. Blood.

[5] Boeckh M, Bowden RA, Goodrich JM, Pettinger M, Meyers JD (1992). Cytomegalovirus antigen detection in peripheral blood leukocytes after allogeneic marrow transplantation. Blood.

[6] Marr KA (2012). Delayed opportunistic infections in hematopoietic stem cell transplantation patients: a surmountable challenge. Hematology Am Soc Hematol Educ Program.

[7] Marr KA, Carter RA, Boeckh M, Martin P, Corey L (2002). Invasive aspergillosis in allogeneic stem cell transplant recipients: changes in epidemiology and risk factors. Blood.

[8] Teira P, Battiwalla M, Ramanathan M, Barrett AJ, Ahn KW, Chen M, Green JS, Saad A, Antin JH, Savani BN, Lazarus HM, Seftel M, Saber W, Marks D, Aljurf M, Norkin M, Wingard JR, Lindemans CA, Boeckh M, Riches ML, Auletta JJ (2016). Early cytomegalovirus reactivation remains associated with increased transplant-related mortality in the current era: a CIBMTR analysis. Blood.

[9] Ljungman P, Engelhard D, de la Cámara R, Einsele H, Locasciulli A, Martino R, Ribaud P, Ward K, Cordonnier C (2005). Vaccination of stem cell transplant recipients: recommendations of the Infectious Diseases Working Party of the EBMT. Bone Marrow Transplant.

[10] Ljungman P, Lewensohn-Fuchs I, Hammarstrom V, Aschan J, Brandt L, Bolme P, Lonnqvist B, Johansson N, Ringden O, Gahrton G (1994). Long-term immunity to measles, mumps, and rubella after allogeneic bone marrow transplantation. Blood.

[11] Parkkali T, Ruutu T, Stenvik M, Kuronen T, Kayhty H, Hovi T, Olander RM, Volin L, Ruutu P (1996). Loss of protective immunity to polio, diphtheria and Haemophilus influenzae type b after allogeneic bone marrow transplantation. APMIS.

[12] Avanzini MA, Locatelli F, Santos CD, Maccario R, Lenta E, Oliveri M, Giebel S, De Stefano P, Rossi F, Giorgiani G, Amendola G, Telli S, Marconi M (2005). B lymphocyte reconstitution after hematopoietic stem cell transplantation: functional immaturity and slow recovery of memory CD27+ B cells. Exp Hematol.

[13] Small TN, Keever CA, Weiner-Fedus S, Heller G, O’Reilly RJ, Flomenberg N (1990). B-cell differentiation following autologous, conventional, or T-cell depleted bone marrow transplantation: a recapitulation of normal B-cell ontogeny. Blood.

[14] Storek J, Witherspoon RP, Storb R (1997). Reconstitution of membrane IgD- (mIgD-) B cells after marrow transplantation lags behind the reconstitution of mIgD+ B cells. Blood.

[15] Storek JFS, Ku N, Giorgi JV, Champlin RE, Saxon A (1993). B cell reconstitution after human bone marrow transplantation: recapitulation of ontogeny?. Bone Marrow Transplant.

[16] Abdel-Azim H, Elshoury A, Mahadeo KM, Parkman R, Kapoor N (2017). Humoral immune reconstitution kinetics after allogeneic hematopoietic stem cell transplantation in children: a maturation block of IgM memory B cells may lead to impaired antibody immune reconstitution. Biol Blood Marrow Transplant.

[17] Gea-Banacloche J, Komanduri KV, Carpenter P, Paczesny S, Sarantopoulos S, Young J-A, El Kassar N, Le RQ, Schultz KR, Griffith LM, Savani BN, Wingard JR (2017). National Institutes of Health hematopoietic cell transplantation late effects initiative: the immune dysregulation and Pathobiology Working Group report. Biol Blood Marrow Transplant.

[18] Mehta RS, Rezvani K (2016). Immune reconstitution post allogeneic transplant and the impact of immune recovery on the risk of infection. Virulence.

[19] Odendahl M, Mei H, Hoyer BF, Jacobi AM, Hansen A, Muehlinghaus G, Berek C, Hiepe F, Manz R, Radbruch A, Dorner T (2005). Generation of migratory antigen-specific plasma blasts and mobilization of resident plasma cells in a secondary immune response. Blood.

[20] Nutt SL, Hodgkin PD, Tarlinton DM, Corcoran LM (2015). The generation of antibody-secreting plasma cells. Nat Rev Immunol.

[21] Stevens RH, Macy E, Morrow C, Saxon A (1979). Characterization of a circulating subpopulation of spontaneous antitetanus toxoid antibody producing B cells following in vivo booster immunization. J Immunol.

[22] D’Orsogna LJ, Wright MP, Krueger RG, McKinnon EJ, Buffery SI, Witt CS, Staples N, Loh R, Cannell PK, Christiansen FT, French MA (2009). Allogeneic hematopoietic stem cell transplantation recipients have defects of both switched and IgM memory B cells. Biol Blood Marrow Transplant.

[23] Greinix HT, Pohlreich D, Kouba M, Körmöczi U, Lohmann I, Feldmann K, Zielinski C, Pickl WF (2008). Elevated numbers of immature/transitional CD21− B lymphocytes and deficiency of memory CD27+ B cells identify patients with active chronic graft-versus-host disease. Biol Blood Marrow Transplant.

[24] Furman D, Jojic V, Sharma S, Shen-Orr SS, Angel CJL, Onengut-Gumuscu S, Kidd BA, Maecker HT, Concannon P, Dekker CL, Thomas PG, Davis MM (2015). Cytomegalovirus infection enhances the immune response to influenza. Sci Transl Med.

[25] Zand MS (2006). B-cell activity of polyclonal antithymocyte globulins. Transplantation.

[26] Roll P, Muhammad K, Stuhler G, Grigoleit U, Einsele H, Tony H-P (2015). Effect of ATG-F on B-cell reconstitution after hematopoietic stem cell transplantation. Eur J Haematol.

[27] Ljungman P, Cordonnier C, Einsele H, Englund J, Machado CM, Storek J, Small T (2009). Vaccination of hematopoietic cell transplant recipients. Bone Marrow Transplant.

[28] Kennedy LB, Li Z, Savani BN, Ljungman P (2017). Measuring immune response to commonly used vaccinations in adult recipients of allogeneic hematopoietic cell transplantation. Biol Blood Marrow Transplant.

[29] Cordonnier C, Labopin M, Robin C, Ribaud P, Cabanne L, Chadelat C, Cesaro S, Ljungman P (2015). Long-term persistence of the immune response to antipneumococcal vaccines after Allo-SCT: 10-year follow-up of the EBMT-IDWP01 trial. Bone Marrow Transplant.

[30] de Masson A, Bouaziz J-D, Le Buanec H, Robin M, O’Meara A, Parquet N, Rybojad M, Hau E, Monfort J-B, Branchtein M, Michonneau D, Dessirier V, Sicre de Fontbrune F, Bergeron A, Itzykson R, Dhédin N, Bengoufa D, Peffault de Latour R, Xhaard A, Bagot M, Bensussan A, Socié G (2015). CD24^high^CD27^+^ and plasmablast-like regulatory B cells in human chronic graft-versus-host disease. Blood.

[31] Sarantopoulos S, Ritz J (2015). Aberrant B-cell homeostasis in chronic GVHD. Blood.

[32] Odendahl M, Jacobi A, Hansen A, Feist E, Hiepe F, Burmester GR, Lipsky PE, Radbruch A, Dorner T (2000). Disturbed peripheral B lymphocyte homeostasis in systemic lupus erythematosus. J Immunol.

[33] MacDonald KP, Blazar BR, Hill GR (2017). Cytokine mediators of chronic graft-versus-host disease. J Clin Invest.

