# Supplementary material for: Measuring the cellular memory B cell response after vaccination in patients after allogeneic stem cell transplantation
Source: Ann Hematol. 2020 Jun 9;99(8):1895–906. doi: 10.1007/s00277-020-04072-9 (PMC7340644; doi:10.1007/s00277-020-04072-9)
Supplement: Supplementary file 2 — (DOCX 62 kb). [file 277_2020_4072_MOESM2_ESM.docx]

**Table S1: Antibodies for flow cytometry**
